# Supplementary material for: iADRGSE: A Graph-Embedding and Self-Attention Encoding for Identifying Adverse Drug Reaction in the Earlier Phase of Drug Development
Source: Int J Mol Sci. 2022 Dec 19;23(24):16216. doi: 10.3390/ijms232416216 (PMC9786008; doi:10.3390/ijms232416216)
Supplement: Supplementary file 1 [file ijms-23-16216-s001.zip › ijms-2081955-supplementary/Supplementary Tables/Supplementary Table S1.pdf]

Table S1. The specific hyper-parameter settings on baseline methods

Baseline	Dropout	Learning_rate	Batch_size	optimizer	Activation
CNN_FP2	0.4	1e-3	64	Adam	RELU
BERT_smiles	0.4	1e-4	64	Adam	RELU
Attentive_FP	0.4	1e-3	64	Adam	RELU
